# Lymphoepithelioma-like carcinoma of the bladder: a case report

**DOI:** 10.3389/fonc.2026.1880826

**Published:** 2026-07-08

**Authors:** Ying Yu, Lei Zhang

**Affiliations:** Department of Urology, Zhejiang Provincial People’s Hospital Bijie Hospital (The First People’s Hospital of Bijie), Bijie, Guizhou, China

**Keywords:** bladder cancer, case report, diagnosis, epirubicin hydrochloride, lymphoepithelioma-like carcinoma, treatment

## Abstract

Lymphoepithelioma-like carcinoma (LELCB), Lymphoepithelioma-like carcinoma of the bladder (LELCB) is a rare subtype of urothelial carcinoma, accounting for less than 1.3% of all pathological types of malignant bladder tumors. Due to the rarity of the cases, surgical resection and postoperative adjuvant therapy are currently the main treatment methods, and standardized treatment protocols and prognostic factors are not yet fully clarified. This article reported a case of an 86-year-old woman who visited the hospital due to painless gross hematuria. Urinary system color Doppler ultrasound and computed tomography (CT) of the bladder both suggested a tumor on the right anterior wall of the bladder. After transurethral resection of bladder tumor, the postoperative pathology confirmed LELCB. The patient relapsed after 11 months of regular chemotherapy. Due to aging and the occurrence of complications such as hypertension, diabetes, and COPD, the patient still chose to undergo partial cystectomy after recurrence. Postoperative pathology still indicated lymphoepithelioma-like carcinoma of the bladder. Unfortunately, the patient did not undergo further adjuvant therapy.

## Background

Lymphoepithelioma-like carcinoma (lymphoepithelioma carcinoma) is a rare undifferentiated malignant tumor closely associated with EBV infection, and its most common site of occurrence is the nasopharynx (1). It occurs very rarely in the urinary system. Urothelial carcinoma has various types, including glandular, squamous, neuroendocrine, and lymphoepithelioma-like (2, 3). LELCB is a rare and aggressive variant of urothelial carcinoma, and it is even less common as a primary bladder tumor. Zukerberg and others first reported primary LELCB of the bladder in 1991 (4). This histological type accounts for about 1% of all malignant bladder tumors. It is worth noting that, unlike lymphoepithelioma-like carcinoma occurring in other sites, LELCB is not associated with EBV infection (5).

We report a case of an 86-year-old female patient who underwent transurethral resection of a bladder tumor and received regular post operative epirubicin hydrochloride chemotherapy. However, the patient still experienced a recurrence 11 months after surgery. Due to its rarity and limited experience, this case adds to the number of treated cases of LELCB, providing potential experience for the treatment of this disease in the future.

## Case presentation

An 86-year-old female patient was admitted to our hospital on May 19, 2025, for treatment due to two months of painless gross hematuria. The patient has a history of ‘rectal polyps’ 5 years ago, has hypertension for 20 years, has no history of smoking, and no family history of tumors. A complete abdominal CT scan without contrast suggests a slightly low-density mass in the right anterior wall of the bladder, approximately 3.0cm × 1.5cm in size (**Figure 1A**). Enhanced scan indicates a slightly low-density mass, with significantly uneven enhancement on the contrast scan, and patchy non-enhancing areas inside, occupying the right anterior wall of the bladder, suggesting the possibility of bladder cancer (**Figure 1B**). After completing the relevant examinations and excluding surgical contraindications, a transurethral resection of bladder tumor (TURBT) was performed on May 28, 2025. Histopathological examination of the postoperative specimen with hematoxylin and eosin (HE) staining revealed tumor cells exhibiting nuclear enlargement, hyperchromasia, irregular nuclear contours, and prominent nucleoli. The tumor cells demonstrated a nested and sheet-like infiltrative growth pattern, invading the lamina propria of the bladder wall. The tumor cells possessed eosinophilic cytoplasm with indistinct cell borders, imparting a syncytial appearance. Mitotic figures were readily identifiable. No definitive necrosis was observed within the tumor tissue. A diffuse and dense lymphoid stromal infiltrate was present surrounding and interspersed among the tumor cell nests, composed predominantly of mature small lymphocytes with scattered plasma cells and histiocytes. The epithelial and lymphoid components were intimately intermingled, together forming the characteristic lymphoepithelioma-like morphology (**Figure 2**). Immunohistochemistry: p63 (+), p53 (mutant type), K5/6 (+), CK7 (-), CK20 (-), CK(Pan) (+), p16 (-), GATA3 (+), Ki-67 (+, 60%). The patient received adjuvant chemotherapy with epirubicin hydrochloride on the first day after surgery. Subsequently, regular chemotherapy was carried out with the following chemotherapy regimen: Epirubicin hydrochloride 50 mg is dissolved in 25 to 50 mL of saline, administered once a week for 8 consecutive infusions; then once a month for 8 consecutive infusions. epirubicin hydrochloride. To prevent the medication from being improperly diluted by urine, patients should refrain from drinking any liquids 12 hours before the infusion. Patients were instructed to void immediately following completion of intravesical therapy.

**Figure 1:**
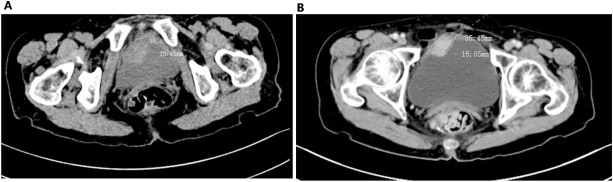
**(A)** Right anterior wall of the bladder a slightly low-density mass-like shadow is seen, approximately 3.0 cm × 1.5 cm in size, with slightly increased density seen inside the bladder. **(B)** Enhanced scan shows obviously uneven enhancement, with patchy non-enhancing areas. A slightly high-density shadow is seen in the bladder, with no enhancement on the enhanced scan.

**Figure 2:**
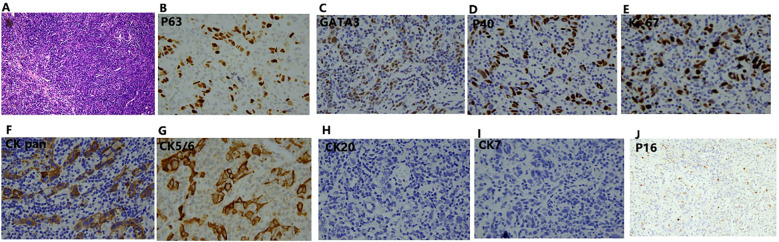
**(A)** Under the microscope, tumor cells are arranged in sheet-like and nest-like patterns, with dense lymphatic infiltration observed in the background and stroma. **(B–J)** Immunohistochemistry (IHC) testing showed: p63 (+), GATA3 (focally weak +), p40 (+), Ki-67 (+, 60%), CK(Pan) (+), CK5/6 (+), CK7 (-), CK20 (-), p16 (-).

However, unfortunately, the patient’s tumor recurred 11 months after surgery. The patient was readmitted on March 31, 2026, due to dysuria. Non-contrast urinary tract CT (**Figure 3A**) and contrast-enhanced CT (**Figure 3B**) revealed tumor recurrence. Concurrent non-contrast chest CT, non-contrast urinary tract CT, and contrast-enhanced pelvic CT showed no evidence of distant metastasis. The patient underwent transurethral resection of bladder tumor (TURBT) on April 16, 2026. Due to the limited extent of surgical resection, the postoperative pathological findings were insufficient to determine the TNM stage or histological grade. The postoperative pathological findings were consistent with the initial pathological diagnosis, both confirming SCNECB. Regrettably, due to the patient’s multiple underlying conditions, further adjuvant therapy is not currently being considered, and up to now, the patient has not undergone further follow-up examinations.

**Figure 3:**
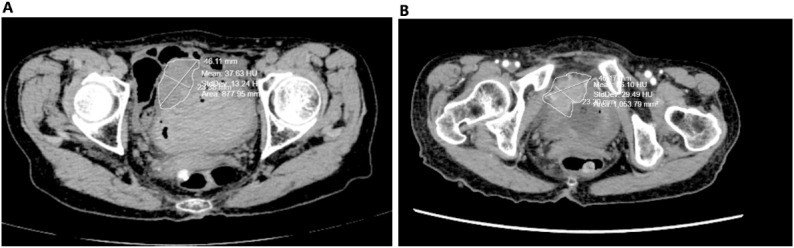
**(A)** (Non-contrast CT): a mass-like, slightly hypodense lesion is observed in the right anterior wall of the urinary bladder, measuring approximately 4.6 cm × 2.3 cm. Slightly increased density and focal gas shadows are noted within the bladder lumen. **(B)** (Contrast-Enhanced CT): A mass-like, slightly hypodense lesion is identified in the right anterior wall of the urinary bladder, measuring approximately 4.6 cm × 2.3 cm. The lesion demonstrates marked heterogeneous enhancement on contrast-enhanced images, with patchy non-enhancing areas within. Slightly hyperdense material is observed within the bladder lumen, which shows no enhancement on contrast-enhanced images; focal intraluminal gas shadows are also noted.

## Follow-up

Since the initial diagnosis of lymphoepithelioma-like urothelial carcinoma of the bladder, the patient has completed 13 months of postoperative follow-up and remains alive as of the last follow-up.

## Discussion

Lymphoepithelioma is an undifferentiated and highly invasive tumor, which was first proposed by Carbone in 1982. Lymphoepithelioma-like carcinoma (LELCB) has histopathological features similar to lymphoepithelioma (6, 7). However, primary LELCB of the urinary tract is very rare. Zukerberg and others first proposed the concept of LELCB in 1991 (4) (**Table 1**). The most common clinical manifestation in patients is painless gross hematuria, which is the main reason for seeking medical attention, and its cause has no obvious connection with common EB virus infection (8). According to relevant literature, it may be potentially related to the high expression of P53 and P63, but the specific mechanism has not yet been clearly studied (9, 10).

**Table 1:** Sex distribution, stage distribution, outcomes, and preferred treatment approaches of LELCB cases.

Author	Year	Age/sex	Subtype	Treatment	Outcome
Alqurashi, Nassar M., et al. (1)	2025	60/M	pT3	TURBT neoadjuvant chemotherapy	Two follow-up cystoscopies showed no evidence of recurrence
Zheng, Gangfu, et al. (6)	2025	70/F	T_2_N_0_M_0_	TURBT	A 25-month follow-up without experiencing a recurrence.
Mitsogiannis, Iraklis, et al. (18)	2022	93/F	–	TURBT	Death occurred three weeks after the start of chemotherapy.
Saini, Swati, et al. (19)	2021	60/M	pT3a	TURBT	Free of disease 8 months after the surgery.
Rahoui, Moez, et al. (20)	2022	52/M	–	Complete transurethral resection of the bladder tumor	No recurrence after 8 months of follow-up
Chaker, Kays, et al. (21)	2018	71/M	–	TURBT	There has been no recurrence after 28 months.
Chen, Chien-Chin, et al. (22)	2017	74/M	–	Radical cystectomy	Died of urinary sepsis 9 months later.
Nagai, Takashi, et al. (8)	2016	43/M	pT3	TURBT	The patient subsequently became free of cancer 72 months postoperatively
Kushida, Nobuhiro, et al. (23)	2015	81/M	T3N1M0	TURBT	He eventually died of other causes 48 months later
Yoshino, Tateki, et al. (24)	2014	83/M	–	TURBT	No recurrence after 12 months of follow-up
Ziouziou, Imad, et al. (25)	2014	64/M	–	TURBT	After 12 months, the patient was in good condition
Yun, Han Ki, et al. (15)	2010	78/F	–	TURBT	The case was followed-up for 8 months without recurrence
Singh, Naorem G., et al. (26)	2009	65/M		Radical cystectomy	Follow-up and is free of symptoms, 12 months after the surgery.
HOLMANG, STEN, et al. (27)	1998	61/F 78/M 65/M 71/F 60/F 65/F 84/F 72/M 71/M	T2 T1 T2 T3 T3 T3 T1 T3 T3	Transurethral bladder resection+ radiation treatment (70 Gy.) Transurethral bladder resection+ radiation treatment (67 Gy.) Transurethral bladder resection Transurethral bladder resection+ radiation treatment (67 Gy.) Transurethral bladder resection+ preop. Radiotherapy andradical cystectomy Transurethral bladder resection Transurethral bladder resection+ preop. Radiotherapy andradical cystectomyTransurethral bladder Resection+ radiation treatment (62 Gy.)	Died of intercurrent diseaseat 18 yrs no autopsy Died of myocardial infarc-tion at 13 mos No blad-der tumor at autopsyNo evidence of disease 24mos. Normal cystoscopy at 21mos died of heart failureat 21 mos No evidence of disease 104mos. No evidence of disease 76mos. Died of disease 66 mos. Died of disease 68 mos. Died of disease 9 mos.

Due to the lack of typical clinical manifestations, patients with LELCB usually present with painless gross hematuria or are found to have a space-occupying lesion of the bladder on urinary ultrasound. Because LELCB lacks characteristic features on ultrasound, imaging examinations, and cystoscopy, it is difficult to differentiate it from common pathological types of bladder tumors using conventional methods; it can only be definitively diagnosed through immunohistochemistry. According to relevant literature, the common immunohistochemical findings of LELCB are: p53 (+), CK3 (+), CK20 (-) (15). This is consistent with the case we reported.

Yang et al. conducted a systematic review of 140 LELCB cases and demonstrated that, based on the proportion of the LELCB component, LELCB can be classified into three subtypes: pure (46%), predominant (26%), and mixed/focal (28%). The pure subtype is associated with a more favorable prognosis (11, 12). Due to the rarity of LELCB, there is no unified treatment protocol for this disease. We reviewed the currently relevant literature (16) and identified the following main treatment options: surgical resection (including TURBT, partial cystectomy, and radical cystectomy) plus postoperative adjuvant therapy (including chemotherapy, radiotherapy, and intravesical chemotherapy). The main chemotherapy regimens are the MVAC regimen (methotrexate + vinblastine + doxorubicin + cisplatin) and the GC regimen (gemcitabine + cisplatin). Based on current reports, although the pure and predominant subtypes are highly invasive, they are also the types with a better prognosis. According to existing research conclusions, patients with pure or predominant LELCB can be treated with bladder-preserving strategies, whereas focal LELCB is more aggressive and necessitates cystectomy. Compared with surgery alone, surgery plus postoperative adjuvant therapy appears to improve patient overall survival (6, 28). In the present case, the diagnosis was LELCB without any concurrent conventional urothelial carcinoma or other variant components. Therefore, this case is classified as the pure type (Pure LELCB). Since no distant metastasis was detected on urinary CT and chest CT, TURBT plus postoperative intravesical instillation therapy was chosen. However, the role of radiotherapy remains unclear.

In recent years, targeted therapy and immunotherapy have emerged as potential treatment approaches. Relevant studies have reported that PD-L1 positivity was observed in 93% of LELCB cases, suggesting potential sensitivity to immune checkpoint inhibitors, making PD-1 therapy a potential treatment option for LELCB patients (13, 14). Another study found that PD-1 was positively expressed in 90% of LELCB tissues through immunohistochemical testing of bladder tissue, providing a theoretical basis for the potential application of immunotherapy in this disease (17). Based on current reports, targeted therapy is indeed a potential and promising treatment option for LELCB.

The overall prognosis of LELCB is relatively poor with a tendency for recurrence, which may be related to the rarity of the disease and the current lack of standardized treatment protocols. Due to the rarity of LELCB, reported survival ranges from 2 months (1) to 68 months (27).

## Limitations

Preoperative imaging evaluation (urological CT and chest CT) revealed no evidence of distant metastasis. The surgery was performed as transurethral resection of bladder tumor (TURBT) rather than radical cystectomy; consequently, the postoperative pathological examination could not yield a complete pTNM staging, and critical parameters including depth of tumor invasion, lymphovascular invasion, and surgical margin status could not be definitively assessed. In addition, EBER *in situ* hybridization was not performed in this study to exclude EBV association, which constitutes one of its limitations. Furthermore, systematic objective follow-up data are lacking, and the 11-month follow-up period is relatively short for evaluating oncological outcomes. We will continue prospective follow-up of this patient and plan to report long-term outcomes (including survival status, recurrence, and subsequent treatment) in the form of an update or correspondence when clinically meaningful events arise in the future.

## Conclusion

This case represents a recurrence of pure LELCB 11 months after the surgery, which differs from previous literature reports. This suggests that there might be an aggressive subgroup within the pure type with high Ki-67 index and p53 mutation characteristics. This discovery provides a new hypothesis for improving the risk stratification system of LELCB. In clinical situations where there is a choice between transurethral resection of bladder tumor (TURBT) for bladder preservation and radical surgery, this case offers a reference for the unfavorable outcome of early recurrence, thereby prompting clinicians to be more cautious in evaluating the pros and cons of bladder preservation treatment.

## Data Availability

The raw data supporting the conclusions of this article will be made available by the authors, without undue reservation.
